# Bibliometric insights into the advancements and future directions of hypofractionated radiotherapy in breast cancer

**DOI:** 10.1007/s12672-026-05140-8

**Published:** 2026-05-05

**Authors:** Wenping Ding, Shuo Liu, Huili Wang, Aibin Zhou, Yihong Chen, Zhenzhen Ye

**Affiliations:** 1 Department of Radiotherapy, The First Affiliated Hospital of Wannan Medical University (Yijishan Hospital of Wannan Medical University), 241001 Wuhu, China; 2 Department of Gerontology, The First Affiliated Hospital of Wannan Medical University (Yijishan Hospital of Wannan Medical University), 241001 Wuhu, China; 3 Department of Pediatrics, The First Affiliated Hospital of Wannan Medical University (Yijishan Hospital of Wannan Medical University), 241001 Wuhu, China

**Keywords:** Breast cancer, Hypofractionated radiotherapy, Bibliometric analysis, VOSviewer, CiteSpace

## Abstract

**Background:**

Radiotherapy is essential after breast cancer surgery to prevent local recurrence. However, conventional fractionation involves extended treatment courses that can strain healthcare resources. In recent years, hypofractionated radiotherapy (HFRT), which delivers larger doses per session over a shorter overall period, has gained traction as a practical and effective alternative. This study employed bibliometric methods to explore the global research landscape and key focus areas in hypofractionated radiotherapy for breast cancer.

**Methods:**

To map out how research in this area has developed, we carried out a bibliometric review of the existing literature. We examined 1,521 English-language studies published between 2005 and 2025, indexed in the Web of Science database. Using tools like Bibliometrix, VOSviewer, and CiteSpace, we tracked publication trends, knowledge frameworks, and collaborative networks over time.

**Results:**

The findings reveal a marked progressive increase in annual publications, with a pronounced upturn after 2020 culminating in a peak of 152 articles in 2021. The United States, Italy, and the United Kingdom contributed the most studies historically, though China has shown striking growth in recent years. The focus of research has shifted: early work centered on optimizing dose schedules, later moving toward assessing toxicity and validating outcomes through clinical trials. Recently, attention has turned to even shorter “ultra-hypofractionated” regimens. Current citation trends highlight toxicity profiling as a particularly active topic of investigation.

**Conclusion:**

Research on HFRT has evolved from establishing non-inferiority to conventional radiotherapy toward a growing focus on safety, precision, and individualized treatment. This study provides a data-driven overview of this evolution, which we hope will help steer future research and inspire further advances in hypofractionated radiotherapy for breast cancer.

## Introduction

 Breast cancer persists as the most frequently diagnosed malignancy in women worldwide, representing a substantial and ongoing global health burden. According to the 2022 Global Cancer Burden Report published by the International Agency for Research on Cancer of the World Health Organization, female breast cancer ranked as the second most commonly occurring cancer globally, with approximately 2.3 million new cases reported. In terms of mortality, the disease was the fourth leading cause of cancer deaths worldwide, accounting for about 666,000 fatalities, which corresponds to 6.9% of all cancer-related deaths [1–5]. Its pathogenesis is marked by considerable molecular heterogeneity, principally influenced by activation of HER2 signaling pathways, hormone receptor status, and pathogenic mutations in genes including BRCA1 and BRCA2 [6]. Although incidence is rising, mortality rates have consistently fallen, largely due to better screening and advances in treatment. Patients with early-stage, non-metastatic disease generally experience favorable outcomes, with current curative-intent approaches achieving success in approximately 70–80% of cases [7, 8]. By contrast, metastatic breast cancer remains incurable with available treatments [9, 10]. Clinical management involves both locoregional and systemic strategies. Locoregional treatments include surgery and radiotherapy, while systemic options consist of endocrine therapy for hormone receptor-positive disease, anti-HER2 agents, chemotherapy, and PARP inhibitors for patients with BRCA1/BRCA2 mutations [11–13]. Despite these advances, major challenges persist. These include reducing racial and ethnic disparities in treatment outcomes, overcoming therapy resistance—especially in aggressive subtypes like triple-negative breast cancer—and improving risk-stratified screening and prevention approaches [14, 15].

Adjuvant radiotherapy plays a crucial role in locoregional treatment, contributing significantly to the reduction of local recurrence and improvement in survival outcomes. Conventional radiotherapy typically involves daily administration over a period of 5 to 6 weeks [16], a schedule that poses considerable challenges for both patients and healthcare infrastructure. These practical limitations have heightened interest in developing more efficient and patient-tolerant approaches. Among these, hypofractionated radiotherapy (HFRT) has garnered increasing attention. By administering a larger dose per fraction, this strategy decreases the total number of treatment sessions, typically allowing treatment to be completed within three to four weeks. Evidence continues to accumulate demonstrating comparable efficacy and safety profiles between hypofractionation and conventional regimens in early-stage disease [17–19]. Its potential application in more advanced stages, such as locally advanced or node-positive disease, remains an active area of research [20, 21]. Even more abbreviated regimens, such as ultra-hypofractionation, are under active clinical evaluation, emphasizing the imperative for robust long-term follow-up data to inform evidence-based guidelines [22–24].

Although numerous studies on hypofractionated radiotherapy have been published, large-scale quantitative analyses tracking the evolution of this field remain limited. A systematic evaluation of publication trends, knowledge frameworks, and collaborative networks is needed to identify research gaps and guide future studies. Bibliometrics provides a powerful suite of methods for such investigations, enabling quantitative characterization of literary growth, key contributing actors, and shifts in research emphasis over time [25, 26].

Therefore, we conducted a bibliometric analysis of hypofractionated radiotherapy for breast cancer based on literature from the Web of Science Core Collection. Using computational tools, we aimed to map the knowledge structure of this field, track its evolution, and pinpoint major and emerging research themes. We also analyzed global collaboration to identify key research networks. This approach offers an objective, data-driven portrayal of the research landscape, which may help guide subsequent studies and support the continued optimization of breast cancer therapy.

## Materials and methods

### Data collection and search strategy

This study employed the Web of Science Core Collection (WoSCC) as the primary data source to systematically retrieve literature pertaining to hypofractionated radiotherapy for breast cancer. To ensure comprehensive coverage and scientific rigor, the search strategy integrated three core thematic domains: breast cancer, radiotherapy, and hypofractionation regimens. A structured query was constructed using synonymous keyword expansions and Boolean logic operators. The final search strategy was formulated as follows: TS=((breast OR “breast cancer”) AND (radiotherap* OR irradiation OR “radiation therapy” OR RT) AND (hypofraction* OR “hypo-fraction*” OR ultrahypofraction* OR “ultra-hypofraction*” OR “short-course” OR “short course” OR “accelerated fraction*” OR “moderate hypofraction*”)).

**Fig. 1:**
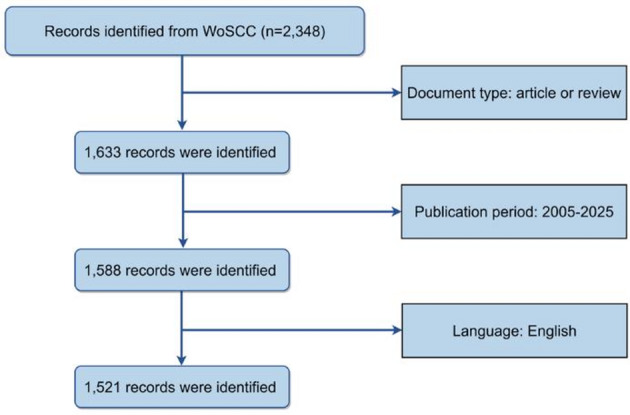
Flowchart of literature search

The literature search was executed on November 16, 2025. As illustrated in Fig. 1, the initial search yielded 2,348 records. After excluding publications inconsistent with the study design criteria, 1,633 articles were retained, comprising 1,364 original research articles and 269 reviews. Further refinement by publication year (2005–2025) resulted in 1,588 articles. Following language restriction (English only), 1,521 articles were included as the final dataset for bibliometric analysis and knowledge mapping. All records were exported in “Full Record and Cited References” format to preserve complete bibliographic metadata, citation data, author affiliations, keywords, funding details, and citation metrics.

### Data analysis and visualization

A multi-software, multidimensional bibliometric analytical framework was adopted to systematically elucidate the research architecture, evolving hotspots, and global collaboration patterns in hypofractionated radiotherapy for breast cancer.

First, all basic statistics were completed using Microsoft Office Excel 2021. These included annual publication trends, literature type distribution, and analyses of research countries and institutions’ contributions. Excel was also used to create basic visualizations such as trend line graphs, bar charts, and pie charts.

The bibliometric analysis was used with the Bibliometrix package (R version 4.3.1) to conduct author collaboration networks, institutional collaboration networks, country collaboration networks, keyword co-occurrence analysis, theme evolution analysis, and burst detection on the 1,521 English articles. The multi-indicator and multi-dimensional analytical framework of Bibliometrix enables a comprehensive analysis of the main thematic areas, researcher collaboration patterns, and evolving trends in hypofractionated radiotherapy research.

In addition to Bibliometrix, VOSviewer (version 1.6.20), ArcGis Desktop (version 10.8.2) and Scimago Graphica (version 1.0.30) were used for knowledge map construction, drawing co-occurrence network diagrams, clustering maps, and heat density maps of authors, institutions, countries/regions, keywords, and references. Its clustering algorithm clearly presents research clusters in the field regarding radiotherapy fractionation methods, biological effects, treatment responses, toxicity management, and technological developments.

Furthermore, CiteSpace (version 6.2.R6) was employed for burst analysis, timeline drawings, citation burst path analysis, and the construction of knowledge evolution tree diagrams. The time-series visualization of CiteSpace enables the identification of key breakthroughs in hypofractionated radiotherapy research across different stages, showcasing the evolution from early discussions on suitable populations to toxicity control, biological dose optimization, and ultimately integration of ultra-hypofractionation with precision radiotherapy technology.

Through the comprehensive application of Excel, Bibliometrix, VOSviewer, and CiteSpace, this study constructed an international knowledge map of hypofractionated radiotherapy research for breast cancer from three dimensions: structure, hotspots, and evolution, laying a solid foundation for subsequent result presentation and trend interpretation.

## Result

### Annual research output and publication trends

**Fig. 2:**
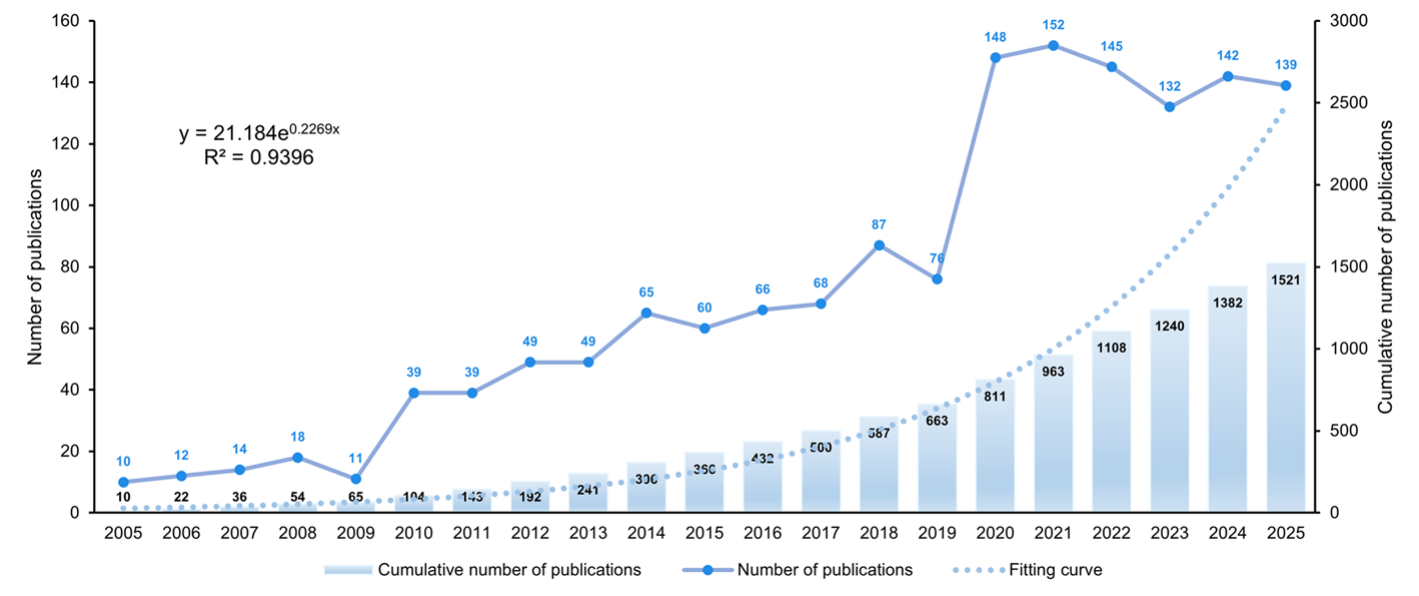
Annual publication volume and cumulative trend from 2005 to 2025. The line indicates annual publication counts, and the bars show cumulative outputs for each year. The dashed line on the right represents an exponential fit (R²=0.9396), suggesting clear exponential growth in the field over time

Analysis of publication trends from 2005 to 2025 demonstrates a characteristic pattern of growth across several phases within the field of hypofractionated radiotherapy for breast cancer (Fig. 2). From 2005 to 2009, research output remained in a nascent stage, with annual publications fluctuating modestly between 10 and 18 articles. This modest growth likely reflects the exploratory nature of early-stage investigations. A noticeable acceleration occurred from 2015 to 2019, as yearly publications rose from 60 to 87. Although a slight dip to 76 articles was observed in 2019, the overall trajectory remained elevated. After 2020, the field expanded rapidly. Publications nearly doubled that year, reaching 148 articles, and hit a peak of 152 in 2021-the highest annual volume during the study period. This sharp increase is likely associated with heightened global interest in the topic, clinical breakthroughs, and impetus from interdisciplinary convergence. From 2023 to 2025, output levels showed minor variations-132, 142, and 139 articles, respectively-though it should be noted that data for 2025 are still incomplete. Despite these fluctuations, the consistently high number of publications indicates that the field has evolved into an established and actively investigated domain, supported by a maturing academic foundation.

### Country/region contributions

**Fig. 3:**
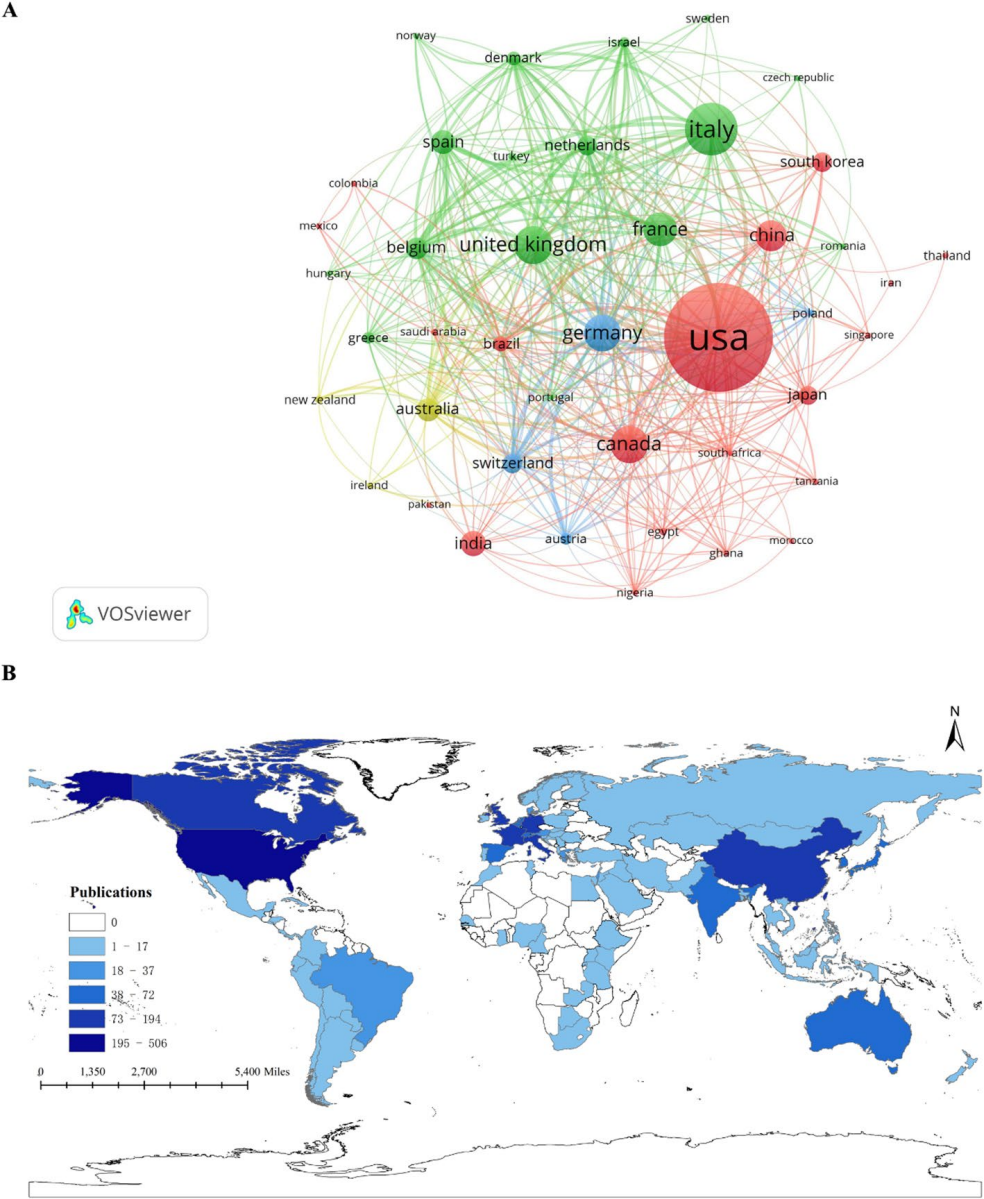
Distribution of countries and collaboration. **A** Country co-occurrence map. Node size indicates publication volume, color represents clustering affiliation, and line density reflects the closeness of cooperation between countries. **B** Country collaboration map. Color represents publication volume

The international collaboration network, as mapped by VOSviewer (Fig. 3A), offers a visual representation of both scientific productivity and the extent of cross-national research partnerships within this domain. The USA prominently occupies a central position in the network and demonstrates high-frequency collaborative relationships with numerous countries. A second group-comprising the United Kingdom, Germany, Italy, Canada, France, and Australia-is also prominent, with these nations forming large nodes that anchor multiple cluster cores. These clusters reflect enduring collaborative networks, particularly between European and North American research entities. At the same time, Asian countries-particularly China, Japan, and South Korea-constitute a vital and rapidly expanding bloc. Although their current node sizes are relatively smaller, the marked increase in their collaborative linkages signals a rapid enhancement of research capacity and integration from this region in recent years.

Assessment of national contributions from 2005 to 2025 delineates a clear hierarchy of global research engagement (Fig. 3B). The United States of America (USA) occupies the foremost position, leading in total publication volume (506 articles), citation count (*n* = 16,422), and strength of international collaborative links (Total link strength = 358), underscoring its pivotal role as a primary knowledge hub. Italy and the United Kingdom follow as significant secondary contributors with 194 and 129 publications, respectively, indicating substantial influence (Table 1).

**Table 1:** Top 10 countries/regions by publication output

Rank	Country	Number of documents	Citations	Total link strength
1	USA	506	16,422	358
2	Italy	194	6936	247
3	United Kingdom	129	12,868	298
4	Canada	124	6214	217
5	Germany	120	6037	222
6	France	108	4444	206
7	China	97	1322	104
8	India	72	485	35
9	Spain	69	1039	161
10	Australia	64	4515	153

### Leading institutions

**Fig. 4:**
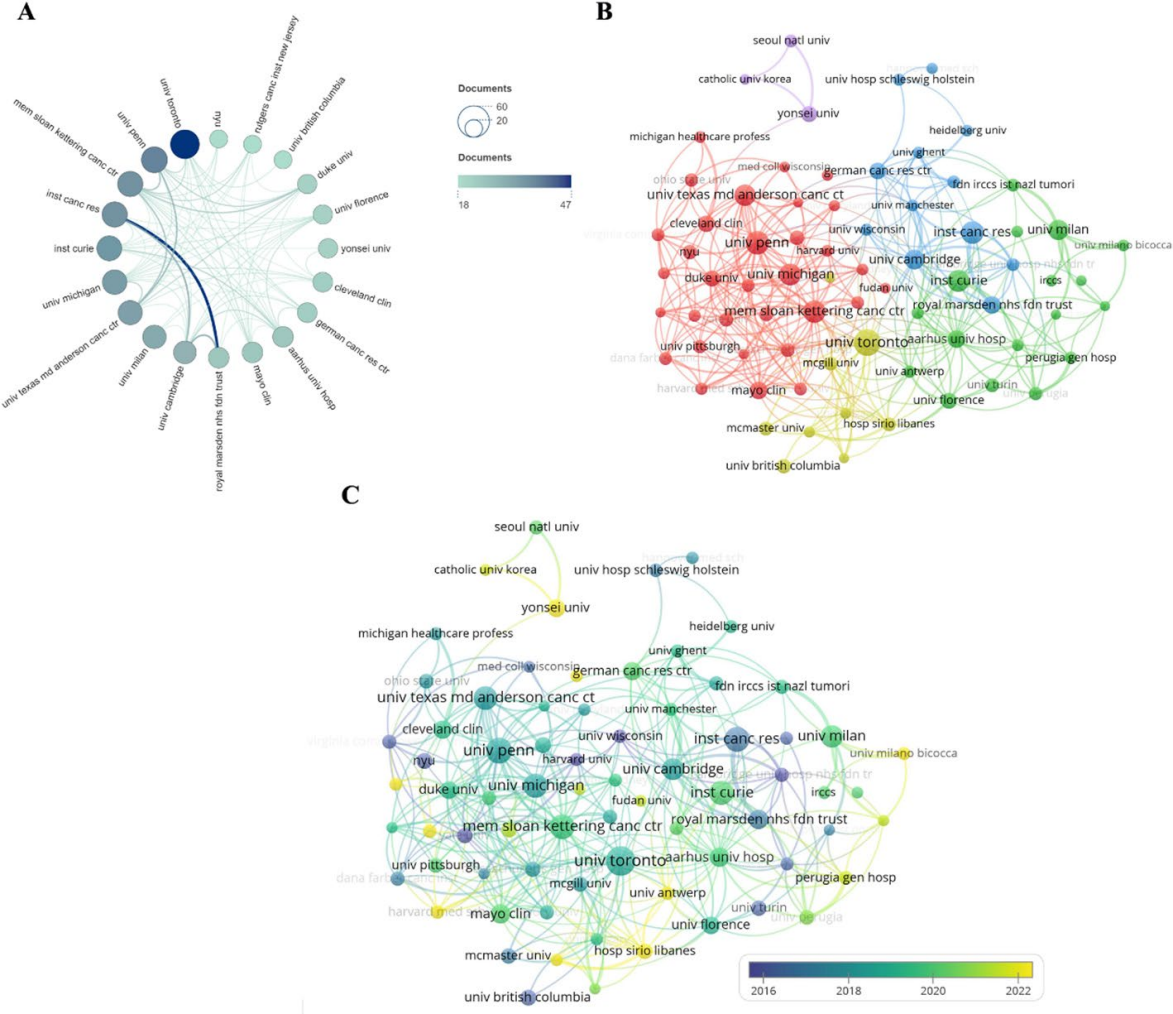
Cooperation network of institutions. **A** Collaboration Network of Top 20 Institutions. The size and color depth of the nodes in the figure represent the number of publications from the institutions. the thickness of the connecting lines represents collaboration intensity. **B** Institution Co-occurrence Map. Node size represents publication volume, color indicates clustering results, and line density and thickness reflect collaboration intensity. **C** Institution Timeline Chart. Node size represents publication volume, and connecting lines reflect collaboration frequency and intensity

Institutional collaboration analysis reveals the foundational architecture of knowledge production in this field. Figure 4A visualizes the collaboration network among the top 20 institutions by publication volume, illustrating the collaborative landscape and the degree of knowledge linkage between different entities. The University of Toronto, represented by its prominent dark blue node at the network’s core, demonstrates a notable advantage in knowledge output within this domain. Furthermore, institutions such as Memorial Sloan Kettering Cancer Center, University of Pennsylvania, Institute of Cancer Research, Institut Curie, and University of Michigan constitute another core community with high collaboration density. Such strong collaborative relationships typically signify long-term team building, shared research platforms, or the joint publication of numerous high-impact studies.

The institution co-occurrence network presented in Fig. 4B further delineates the patterns of collaborative frequency and associated knowledge flows. A prominent and tightly connected red cluster is evident, centered on leading USA institutions. Key hubs within this cluster include the University of Texas MD Anderson Cancer Center, Memorial Sloan Kettering Cancer Center, and Harvard University, with strong participation from the University of Pennsylvania, Duke University, and the Cleveland Clinic. The density of linkages here points to robust and sustained domestic partnerships, forming what appears to be the most cohesive core for knowledge generation in the field. A distinct green cluster represents a European institutional alliance, including the University of Milan, Institut Curie, Royal Marsden NHS Foundation Trust, and IRCCS (Istituto di Ricovero e Cura a Carattere Scientifico). This cluster is internally well-connected and features several cross-regional collaborative bridges to the red cluster.

As shown in Fig. 4C, this network depicts the evolution of institutional activity and collaborative structures across different phases. Central to this network are USA institutions, which maintain a dominant presence. Key nodes such as the University of Texas MD Anderson Cancer Center, Memorial Sloan Kettering Cancer Center, and the University of Pennsylvania, alongside the University of Michigan, are notably large and appear across multiple yearly colors (2016–2022). This pattern underscores their sustained productivity and central role in the collaborative network throughout the period examined. European institutions like the University of Cambridge, Institut Curie, University of Milan, and German Cancer Research Center are distributed on the right side of the network. Their node colors transition from green to yellow, suggesting a notable rise in research activity between 2018 and 2022. Asian institutions, including Yonsei University, Seoul National University, and The Catholic University of Korea, are represented primarily by green or yellow nodes (2019–2022), reflecting the recent, gradual emergence of East Asian research strength.

Table 2 shows that the institution with the highest publication volume is the University of Toronto (*n* = 47). The University of Pennsylvania, the Institute of Cancer Research, and Memorial Sloan Kettering Cancer Center follow with 38, 36, and 36 publications, respectively. The institution with the highest citation count is the Institute of Cancer Research (*n* = 4,052).

**Table 2:** Top 10 institutions by number of publications

Rank	Institution	Number of documents	Citations	Total link strength
1	University of Toronto	47	1561	63
2	University of Pennsylvania	38	2357	87
3	Institute of Cancer Research	36	4052	57
4	Memorial Sloan Kettering Cancer Center	36	1844	101
5	Institut Curie	35	693	43
6	University of Michigan	34	2093	52
7	University of Texas MD Anderson Cancer Center	33	2068	68
8	University of Milan	31	487	54
9	University of Cambridge	29	1989	77
10	Aarhus University Hospital	24	749	67

### Prominent authors

**Fig. 5:**
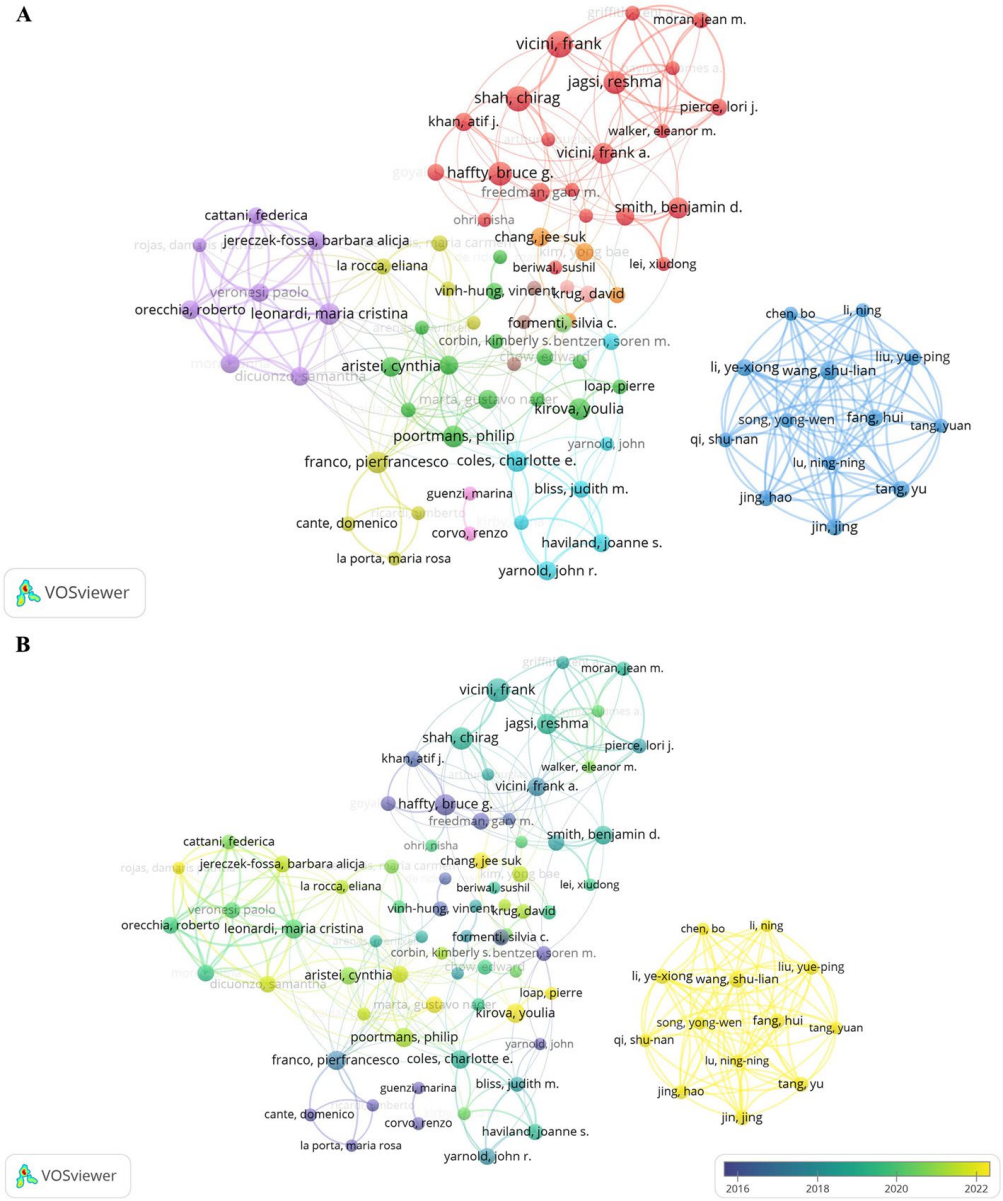
Collaboration network of authors. **A** Author co-occurrence network. Node size indicates publication volume, connections represent co-authorship frequency, and node color indicates different collaborative clusters. **B** Author timeline map. Node color shifts from purple (2016) to yellow (2022). Node size corresponds to publication count, and connecting lines indicate co-occurrence relationships

The author co-occurrence network map illustrates the collaborative density, academic connections, and research group structures among different investigators within the same thematic field. As depicted in Fig. 5A, the network exhibits a distinct multi-centric, transnational collaborative pattern. The red cluster positioned at the top represents a leading North American research group in breast radiotherapy—including researchers like Vicini F, Jagsi R, and Haffty BG, features large nodes and dense interconnections, reflecting solid internal collaboration and a highly concentrated research focus. The blue cluster in the lower-right section constitutes a Chinese research alliance, including authors such as Li N, Chen B, Li YX, and Wang SL. The highly dense internal links within this cluster form a typical domestic collaborative network. The green cluster represents a broad European collaboration network centered around researchers like Poortmans P, Aristei C, and Kirova Y, characterized by a high degree of multinational cooperation.

The author timeline network map shown in Fig. 5B reveals the temporal rhythm of researcher involvement and the evolutionary paths of research teams. From 2016 to 2018, a cluster of purple-blue nodes primarily revolved around European researchers like Aristei C and Poortmans P, who were the main driving forces in international breast radiotherapy research at that time. Between 2018 and 2020, a notable increase in green nodes marks the peak activity period of the North American-led team, represented by Vicini F, Jagsi R, and Haffty BG. From 2020 to 2022, a dense concentration of yellow nodes emerged in the lower-right area of the map, signifying the rapid rise of Asian teams, predominantly from China (e.g., Li N, Chen B, Fang H, Tang Y), in recent years. This timeline reflects how research strengths from different regions have assumed knowledge-propelling roles at different stages, illustrating the domain’s typical globalized relay-style development pattern.

Table 3 lists the top 10 authors by publication count. The author with the highest output is Vicini F (*n* = 23), followed by Shah C and Haffty BG with 21 and 20 publications, respectively. The author with the highest citation count is Coles CE (*n* = 1,866).

**Table 3:** Top 10 authors by number of publications

Rank	Author	Number of documents	Citations	Total link strength
1	Vicini F	23	641	31
2	Shah C	21	419	28
3	Haffty BG	20	730	25
4	Jagsi R	19	1226	30
5	Franco P	17	544	13
6	Kirova Y	17	68	2
7	Leonardi MC	17	183	88
8	Poortmans P	17	722	23
9	Coles CE	16	1866	29
10	Smith BD	16	1132	16

### Journal analysis

**Fig. 6:**
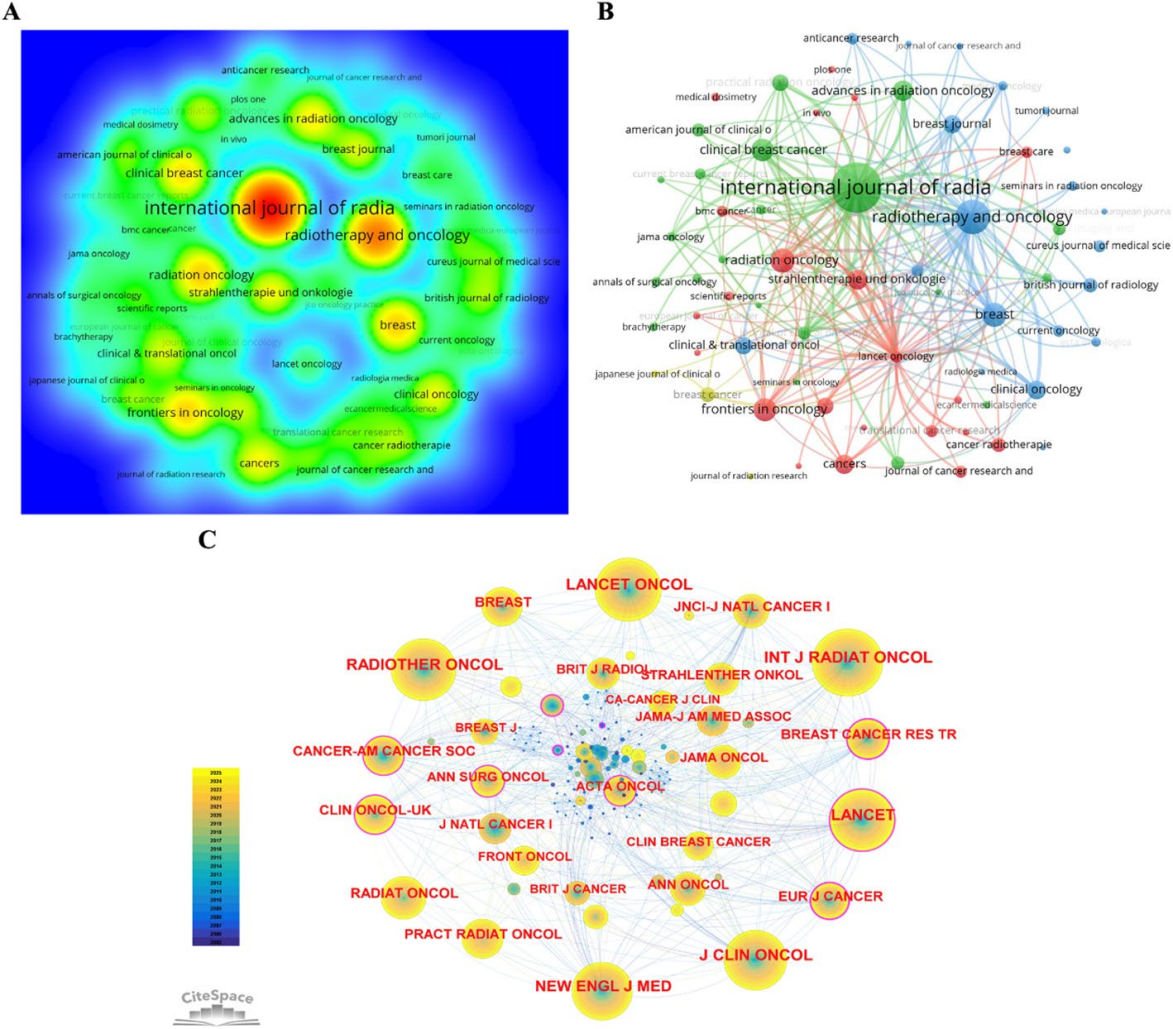
Publication volume of productive journals.** A** Journal density map. The colors gradient from blue (low density) to red (high density). **B** Journal co-occurrence map. Node size indicates publication volume, and connections represent collaborative relationships or common focuses on themes. **C** Journal co-citation network. Node size represents the frequency of co-citation, and color reflects the temporal gradient (blue for early, yellow for recent)

The journal density map presented as a heatmap depicts the publication hotspots and concentration levels of different journals within the research field (Fig. 6A). At the center of the map lies a concentrated red region—primarily formed by the *International Journal of Radiation Oncology Biology Physics* and *Radiotherapy and Oncology*. This suggests these two journals are the main places where research is published most densely, making them key knowledge hubs in this field. Adjacent to the red zone are yellow and green areas. Journals such as *Clinical Breast Cancer*, *Frontiers in Oncology*, and *Cancers* form regions of relatively high density, reflecting notable contributions from breast cancer-specific research, comprehensive oncology studies, clinical prognosis, and imaging to the field. The outer blue areas are composed of smaller or interdisciplinary journals, indicating their participation in the ecosystem but lower thematic centrality.

The journal co-occurrence network shown in Fig. 6B reflects the frequency with which different journals appear together on the same research topics. The largest node corresponds to the *International Journal of Radiation Oncology*,* Biology*,* Physics*, confirming its role as the primary publishing platform covering diverse themes such as radiotherapy techniques, clinical trials, and dosimetric evaluation. The blue cluster, including *Radiotherapy and Oncology*, comprises journals primarily focused on radiotherapy techniques and imaging management, highlighting their shared contribution to methodological research and image-guided strategies. The red cluster centers around comprehensive oncology journals such as *Frontiers in Oncology*, *Cancers*, and *Lancet Oncology*. Their thematic orientation leans more towards comprehensive oncology, clinical trials, and interdisciplinary research, demonstrating their notable role in studying breast cancer, radiotherapy toxicity, and prognostic factors.

The journal co-citation network within the field is illustrated in Fig. 6C. In terms of node size, *Lancet Oncology*, *International Journal of Radiation Oncology*,* Biology*,* Physics*, and *Radiotherapy and Oncology* constitute the three largest nodes, confirming their status as primary platforms for knowledge dissemination and the aggregation of clinical evidence. Comprehensive clinical medicine journals, such as *The New England Journal of Medicine* and *Journal of Clinical Oncology*, also appear as large nodes, reflecting the major impact of the clinical trial results they publish on radiotherapy strategies and long-term patient survival outcomes. Prominent nodes such as *Clinical Breast Cancer* and *Breast Journal* indicate that specialized journals have established a consistent group of contributors in the field of breast cancer. From the figure, we can see a clear three-tier path for how knowledge spreads in this field: it flows from leading clinical journals, to top specialty journals, and then to more technical publications.

### Reference analysis

**Fig. 7:**
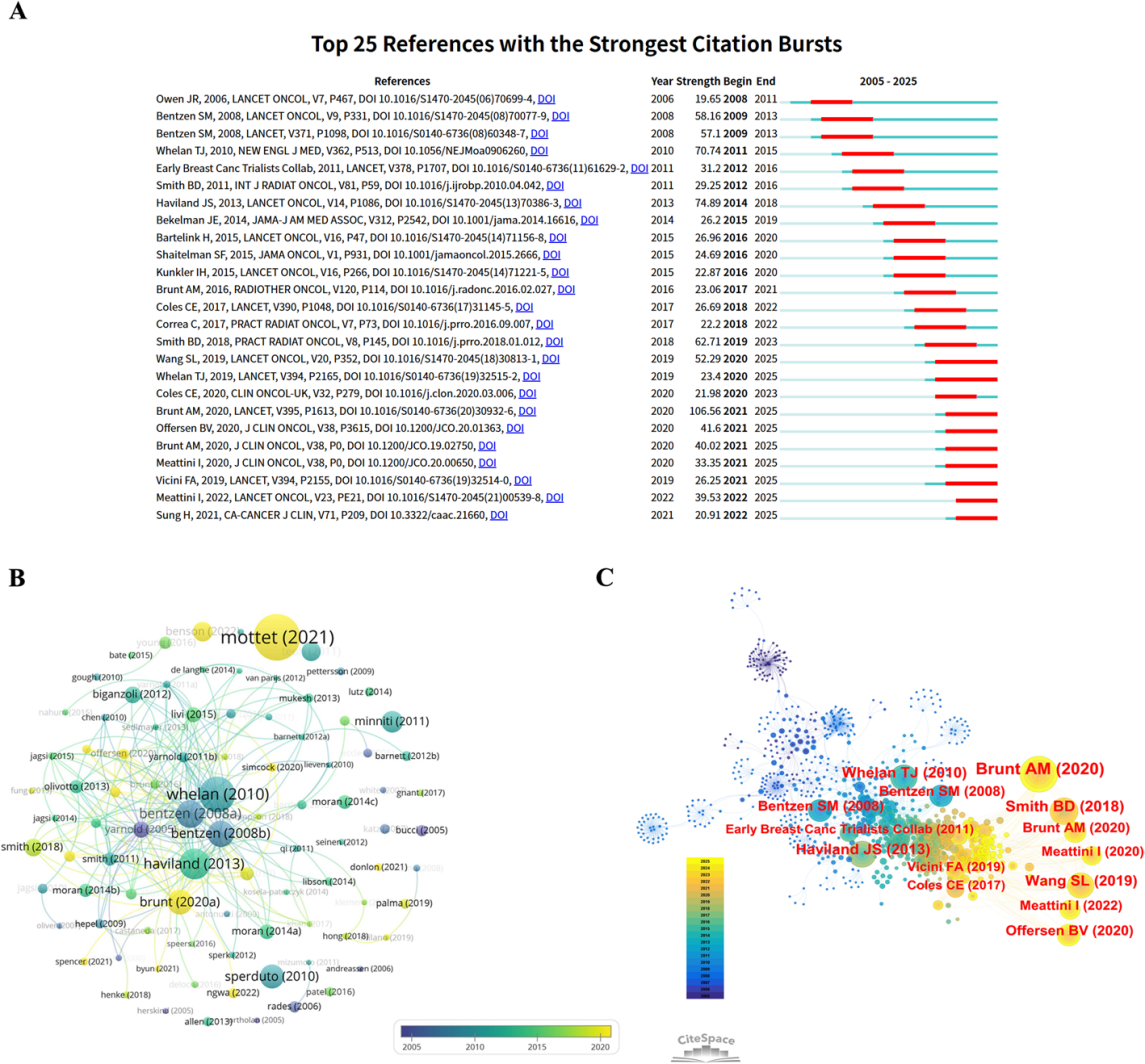
Analysis of cited references. **A** References with the strongest citation bursts. The horizontal axis represents the time range of burst occurrence (2005–2025), with the red interval indicating the duration of the burst, and the blue-green segments marking the background citation volume before and after the burst. **B** Citation timeline network. Node size indicates citation frequency, color represents publication year (deep purple for 2005 and bright yellow for 2020), and connections show co-citation relationships among literature. **C** Literature co-citation network. Node size represents the frequency of literature being cited, node color indicates the year of publication (deep blue/purple for early, yellow for recent), and connections display the frequency of literature co-occurring in reference lists

Figure 7A displays the top 25 references with the strongest citation bursts between 2005 and 2025. The early phase (2005–2011) featured a number of foundational classic publications, such as Owen JR et al. (2006), Bentzen SM et al. (2008), and Whelan TJ et al. (2010), with burst strengths of 19.65, 58.16, and 70.74, respectively. During the period of 2012–2016, the number of bursting references continued to increase, forming a second peak of research attention. Representative publications included Haviland JS et al. (2013), Smith BD et al. (2011), and Bartelink H et al. (2015). After 2017, a clear thematic shift became evident, with bursts increasingly concentrated on innovative technologies. Notably, multiple strong burst references emerging post-2020 continued through 2025, such as Whelan TJ et al. (2019), Offersen BV et al. (2020), and Meattini I et al. (2020).

The citation timeline network, presented in Fig. 7B, further elucidates the evolutionary trajectory of the research knowledge base. In the central region, Bentzen SM et al. (2008) and Whelan TJ et al. (2010) form the most central knowledge nodes. Surrounding these, mid-period publications like Haviland JS et al. (2013), Moran et al. (2014), and Olivotto et al. (2013) form dense network clusters. Post-2018, nodes expand outward, representing the latest highly cited literature, such as Smith et al. (2018), Brunt et al. (2020), Wang SL et al. (2019), and Meattini et al. (2020, 2022). In the peripheral areas of the graph, we see several large, yellow-colored nodes, such as those for Mottet et al. (2021). Their appearance suggests these recent publications are being cited quickly, marking them as new and growing areas of interest in the field. These publications often focus on frontier topics like ultra-hypofractionation, toxicity analysis, regional nodal irradiation, and surgical technique variations, reflecting the field’s accelerated advancement towards treatment refinement and personalized strategies.

The document co-citation network, constructed to reveal the knowledge base and core literature lineage of the field, is shown in Fig. 7C. On the left, nodes mainly in deep blue-like Bentzen SM et al. (2008)-are early foundational studies. The central area, featuring nodes in green to yellow, corresponds to key clinical studies published between 2010 and 2017. Notably, the large nodes for Whelan TJ et al. (2010) and the Early Breast Cancer Trialists’ Collaborative Group (2011) represent pivotal randomized controlled trials that defined radiotherapy strategies and validated fractionation schedules. On the right, the large yellow nodes stand out—these are recent highly-cited papers from 2018 to 2022, with publications such as Brunt AM et al. (2020), Meattini I et al. (2020, 2022), and Offersen BV et al. (2020) significantly enlarged.

### Keyword analysis

**Fig. 8:**
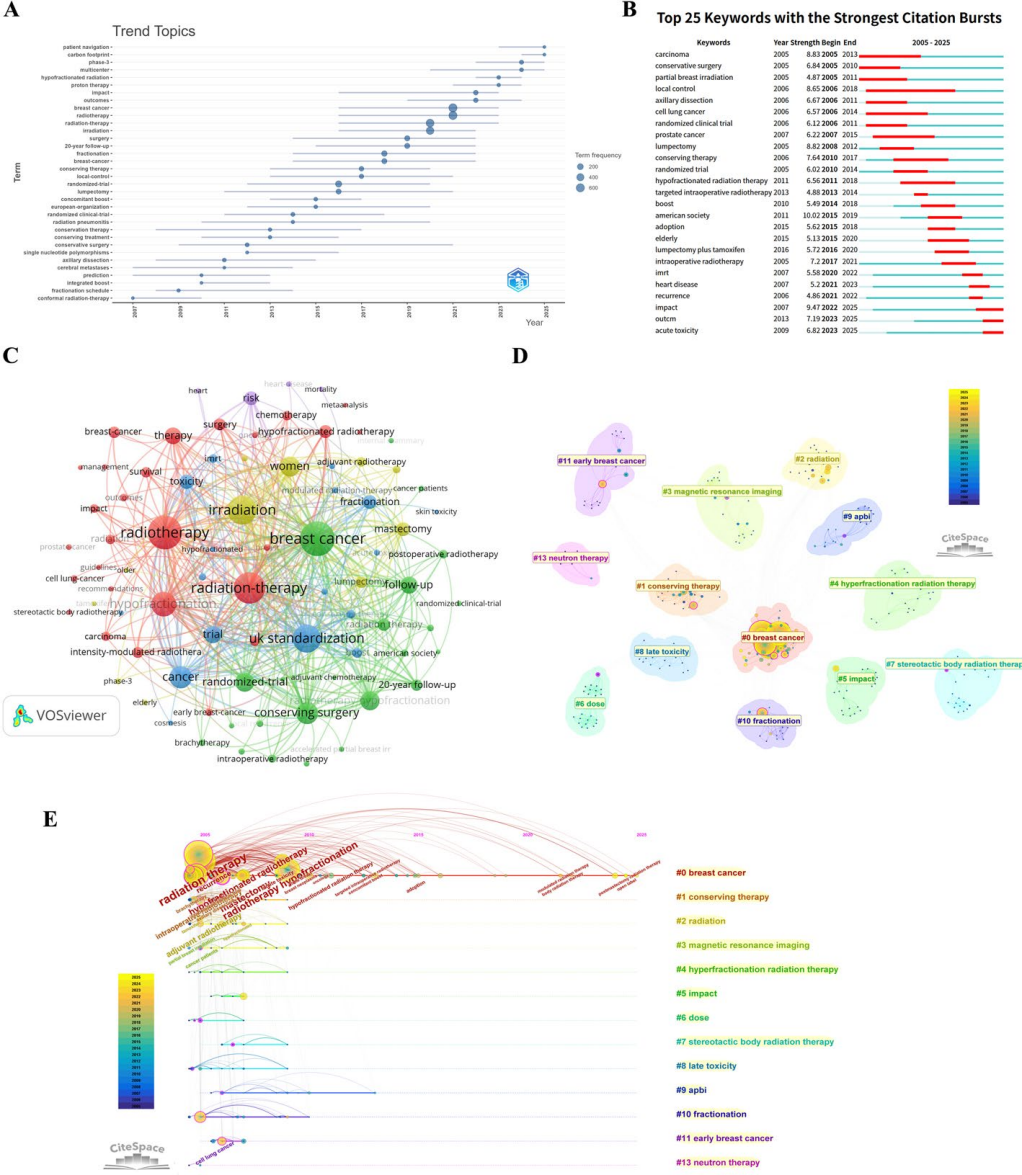
Cluster analysis and topic evolution of keywords. **A** Keyword trend chart. The bubble size is proportional to the frequency of keyword occurrence. The horizontal axis represents the year, while the vertical axis lists the specific terms. Each horizontal bar corresponds to a keyword and visually indicates its “active year span”-the period during which the term sustained notable attention in the literature. **B** Top 25 keywords with strongest citation bursts. Each keyword corresponds to its burst intensity, start and end years, and the burst time interval (indicated in red). **C** Keyword co-occurrence map. The node size is proportional to the frequency of keyword occurrences, and the node colors reflect different clustering groups, while the connecting lines indicate the frequency and strength of co-occurrence of keywords within the same literature. **D** Keyword clustering map. Each cluster represents a relatively independent thematic group, with different background colors representing different clustering modules; the size of the nodes indicates the frequency of keyword occurrences, and the connections between nodes reflect the strength of semantic coupling. **E** Keyword clustering timeline. The horizontal axis represents the years, while the vertical axis displays the cluster numbers. Each point in the cluster represents critical nodes appearing in the literature, with colors reflecting the emergence time, and arcs indicating citation associations among keywords

The trend evolution of keywords based on their temporal distribution is depicted in Fig. 8A. Early terms (2007–2012), such as “conformal radiation-therapy,” “fractionation schedule,” “prediction,” and “cerebral metastases,” were primarily focused on basic treatment standards and exploratory pre-/intra-operative strategies. The period from 2012 to 2017 represented a peak phase of technological innovation, marked by the sustained presence of specialized terms like “randomized clinical trial,” “local-control,” “conserving therapy,” “20-year follow-up,” and “hypofractionated radiation.” Between 2017 and 2025, the field entered an expansion and inquiry phase, with the rapid emergence of terms like “proton therapy,” “phase-3,” “multicenter,” and “carbon footprint,” illustrating the expansion of research into broader socio-technical domains.

The keywords with strongest citation bursts aim to identify the focal points of research attention and their evolution across different time periods (Fig. 8B). The early stage (2005–2011) was dominated by basic clinical terminology such as “carcinoma,” “conservative surgery,” “partial breast irradiation,” “local control,” and “axillary dissection,” reflecting the initial research focus on disease diagnosis, tumor characteristics, local control strategies, and traditional surgical methods. The mid-stage (2011–2018) witnessed more specialized and nuanced burst keywords, including “hypofractionated radiation therapy,” “targeted intraoperative radiotherapy,” “boost,” and “american society,” representing the development of specific technical pathways and guidelines. The late stage (2020–2025) shows a distinct thematic shift, with burst keywords highly concentrated on “impact,” " outcome “, “heart disease,” and “acute toxicity.” This vocabulary indicates an evolution of the research paradigm from technological innovation towards clinical outcome optimization, long-term prognosis, and comprehensive toxicity management.

Figure 8C displays the semantic structure within the research domain and the co-occurrence logic between themes. Keywords such as “radiotherapy,” “breast cancer,” “irradiation,” and “radiation therapy” occupy central positions with large node sizes, indicating they are not only foundational concepts but also major connecting points for various thematic branches. Different thematic clusters form around these cores. For instance, a cluster dominated by green nodes like “breast cancer-follow-up-mastectomy-local control-conserving surgery” focuses on surgical and long-term outcome aspects. A cluster with red nodes centered on “radiotherapy-toxicity-therapy-outcomes” emphasizes treatment efficacy, therapeutic response, and the management of adverse effects. In terms of network density, significant coupling is observed between technical terms like “hypofractionated radiotherapy” and “stereotactic body radiotherapy” and clinical outcome-related keywords like “toxicity” and “follow-up,” indicating that current research is transitioning from technological innovation towards comprehensive efficacy evaluation.

Further cluster analysis of this network summarizes the main and evolving research directions (Fig. 8D). Cluster #0 “breast cancer” acts as the primary central hub, connecting other thematic threads. Clusters #1 “conserving therapy” and #2 “radiation” form the main framework of radiotherapy strategy research. More specialized clusters, including cluster #4 “hyperfractionation radiation therapy,” cluster #7 “stereotactic body radiation therapy,” and cluster #3 “magnetic resonance imaging,” represent distinct directions such as technical optimization, high-precision delivery, and image guidance, respectively.

The timeline distribution of these themes further illustrates their evolutionary trajectories, as shown in Fig. 8E. The largest and most stable cluster, #0 “breast cancer,” spans the entire period from 2005 to 2025, representing the enduring core theme. Cluster #1 “conserving therapy,” active between 2007 and 2018, reflects the process by which breast-conserving therapy evolved into a standard clinical pathway. Technology-focused clusters, such as cluster #4 “hyperfractionation radiation therapy” (emerging around 2010) and cluster #7 “stereotactic body radiation therapy,” show patterns of later emergence and concentrated growth, consistent with technological advancements. Meanwhile, safety-oriented clusters like cluster #8 “late toxicity” (highly active from 2012 to 2022) indicate an expansion of the research scope from pure efficacy to a dual focus on “efficacy and safety.”

## Discussion

Advances in radiotherapy techniques have spurred growing interest in HFRT as a promising alternative to standard conventional fractionation for breast cancer [27, 28]. The use of HFRT in breast cancer is supported by an evolving radiobiological understanding, particularly the premise that breast cancer cells may have a relatively low α/β ratio [29, 30]. This suggests they are more sensitive to higher doses per fraction compared to many early-responding healthy tissues. This forms the theoretical basis for reducing overall treatment duration while escalating dose per fraction [31–33]. The development of HFRT has not followed a straightforward path. Early challenges primarily involved limited long-term safety data and insufficient evidence regarding its non‑inferior efficacy [34]. Clinical concerns have focused on whether larger fraction sizes might raise the risk of late toxicities-such as fibrosis, lymphedema, and notably cardiotoxicity, which is especially relevant for left‑sided breast cancer. Consequently, a key ongoing research effort aims to optimize fractionation schedules while maintaining rigorous long‑term toxicity surveillance [35, 36]. Moreover, translating radiobiological principles from bench to bedside into widely applicable clinical protocols remains challenging, particularly when accounting for factors such as disease stage, type of surgery (e.g., breast-conserving or mastectomy), and variations in individual patient anatomy [37, 38]. Clinical trials have shown that HFRT, which delivers greater doses per fraction over a shorter period, achieves comparable efficacy and toxicity to conventional radiotherapy, while offering potential benefits in patient convenience and healthcare efficiency [39, 40]. This bibliometric study maps the research landscape of HFRT for breast cancer by analyzing the relevant literature published from 2005 to 2025.

The results of this study-ranging from broad publication trends and collaboration patterns down to specific shifts in research themes-provide empirical support for the recognized phases of development and ongoing challenges within the field of hypofractionated radiotherapy for breast cancer. Annual publication trends align closely with the distinct phases of evidence generation. In the initial 2005–2009 period, characterized by relatively low output, research mainly comprised preliminary exploratory and prospective studies that assessed the fundamental safety and feasibility of hypofractionated regimens [41, 42]. Following 2015, the marked rise in publications coincides with the release and longer-term follow‑up data from key large‑scale randomized controlled trials. These studies provided robust, high‑level evidence demonstrating non‑inferior efficacy for hypofractionated regimens [43, 44]. The sharp increase in publications after 2020, peaking in 2021, reflects a new phase of research intensity as HFRT became widely adopted in clinical practice. With this shift, the focus of studies has moved from establishing basic feasibility to refining treatment protocols and identifying which patient subgroups benefit most [45, 46].

Changes in the contributions by country/region and the patterns of collaboration reflect the evolving geography of knowledge production. The central, dominant role of the United States in the network stems from its early investment in large‑scale clinical studies, leadership in pivotal trials, and substantial output from leading cancer centers. European nations contributed early work on methodological aspects, including dose planning, normal‑tissue constraints, and quality‑of‑life assessment. A notable trend is the rapid growth in research output from Asian countries, especially China, and their quick integration into international collaborative networks since around 2015. This not only expands the global evidence base but also stimulates studies focused on the anatomical and epidemiological characteristics of Asian populations, often providing new perspectives on the generalizability of treatment protocols [47, 48].

Analyzing the research community and its publication patterns reveals the intellectual structure of this field. The emergence of distinct author clusters-such as North American groups leading technology translation [49, 50], European teams specializing in toxicity management and long-term follow-up [51, 52], and recently prolific Chinese researchers conducting large-scale clinical studies [53–55]-collectively functions as a multi-engine propelling the field forward. This transition from early European leadership, through a period of North American dominance, and later the rise of Asian contributions, highlights not only shifts in research capacity but also a significant geographical expansion in the field’s investigative focus. Regarding knowledge dissemination, specialized journals and high-impact general medical publications form a complementary system. The former offer a platform for advancing technical and methodological details, whereas the latter play a key role in disseminating results from major trials that influence practice, thereby helping shape clinical guidelines and promote the uptake of consensus-based standards of care [56–58].

Examining the knowledge base and research fronts offers a clearer view of how this field has evolved. Through methods such as reference burst detection and co-citation analysis, three major phases of evidence development can be traced. The early phase includes foundational studies that established key radiobiological principles [59–62]. The mid-phase consists of randomized trials that provided critical evidence supporting the adoption of standard-of-care practices [63–66]. The most recent phase focuses on emerging approaches such as ultra-hypofractionation and intraoperative radiotherapy [67–72]. This progression follows the classic development path for a medical intervention, moving from theoretical groundwork to empirical confirmation and then to broader exploratory research. At the same time, shifting keyword usage reveals a notable change in research priorities. Early work centered on technical endpoints such as “local control” and “conserving surgery”; the mid-period emphasized “fractionation schedule” and “clinical trial”; more recently, bursts in keywords like “outcome,” “heart disease,” “acute toxicity,” and “impact” have emerged. This evolution suggests a growing emphasis on long-term quality of life, late effects-particularly cardiovascular toxicity-and overall treatment impact [73–75], directly addressing the long‑standing challenge of balancing efficacy with safety. Moreover, The appearance of terms such as “carbon footprint” further signals that the field is expanding to incorporate broader socio‑environmental considerations. Taken together, these trends point to a multidimensional research framework spanning the disease itself (breast cancer), treatment modality (radiotherapy), technical refinement (fractionation), clinical outcomes (toxicity, follow‑up), and research methods (randomized trials). This framework helps clarify the major research themes and their interconnections.

This study is subject to several limitations, chiefly concerning our data sources. Our analysis relied solely on the Web of Science Core Collection, which, while providing high-quality and standardized data, may not capture all relevant publications, particularly those in non-English journals or regional databases. It is also important to note that the literature search was performed in November 2025, so the data for that year are likely incomplete. Furthermore, the bibliometric techniques employed are subject to inherent biases. For instance, the detection of non-English keywords can be limited, potentially leading to an underrepresentation of contributions from non-English-speaking regions. Another constraint is the absence of patent data, which limits insights into how technological developments are being translated into clinical and commercial applications. These constraints highlight the importance of using more diverse and inclusive data sources in future bibliometric studies to better capture advancements in hypofractionated radiotherapy.

## Conclusions

This bibliometric analysis maps the evolution of hypofractionated radiotherapy in breast cancer over the past two decades. The findings highlight its emergence as an integrative field spanning imaging technology, dosimetric innovation, and clinical oncology. The growing volume and global collaborative nature of the literature underscore its increasing relevance. Looking ahead, two research priorities stand out: generating robust long-term toxicity data for ultra-hypofractionated regimens, and incorporating artificial intelligence into personalized treatment planning. Progress in these areas will likely enhance patient outcomes and further solidify hypofractionated radiotherapy as a cornerstone of modern oncology. 

## Data Availability

The datasets generated and/or analyzed during the current study are available in the Web of Science Core Collection (WoSCC) database, URL: https://www.webofscience.com/wos/woscc/basic-search. Further inquiries can be directed to the corresponding authors.
